# Supplementary material for: Pupil size predicts exploration through critical slowing in prefrontal dynamics
Source: Commun Biol. 2025 Dec 20;9:103. doi: 10.1038/s42003-025-09372-2 (PMC12830900; doi:10.1038/s42003-025-09372-2)
Supplement: Supplementary file 2 — Supplementary information [file 42003_2025_9372_MOESM2_ESM.pdf]

Title:

Pupil size predicts exploration through critical slowing in prefrontal dynamics

Authors:

Akram Shourkeshti[1†], Mojtaba Abbaszadeh[1†], Gabriel Marrocco[1], Katarzyna Jurewicz[1,2], Tirin Moore[3,4], R. Becket Ebitz*[1]

Affiliations:

[1]Department of Neurosciences, Université de Montréal, Montréal, QC, Canada

[2]Department of Physiology, McGill University, Montréal, QC, Canada

[3]Department of Neurobiology, Stanford University School of Medicine, Stanford, CA, USA

[4]Howard Hughes Medical Institute, Chevy Chase, MD, USA

† These authors contributed equally to this work.

*Corresponding author and lead contact:

R. Becket Ebitz

Department of Neurosciences

Université de Montréal

Montréal, QC CANADA H3T 1J4

Email: becket@ebitzlab.com

Supplementary Table 1

Regression coefficients and p values for the mediation analysis testing whether the scatter index mediates the relationship between pupil size and the onset of exploration. Related to **Figure 4J**.

		$pupil_{t-1}$ → $scatter_{t-1}$ → $explore_t$	
		est. coefficient	p value <
Total effect	c	0.090	0.005
Effect on mediator	a	0.036	0.0005
Unique mediator effect	b	0.092	0.005
Indirect effect	ab	0.003 (z = 2.70*)	0.005
Direct effect	c’	0.086	0.005
Sample size	n = 28 sessions, 21,425 trials		

*Sobel’s test

Supplementary Table 2

Regression coefficients and p values for the mediation analysis testing whether response time slowing mediates the relationship between pupil size and the onset of exploration on the next trial. Related to **Figure 5C**.

		$pupil_{t-1}$ → RT $slowing_{t-1}$ → $explore_t$	
		est. coefficient	p value <
Total effect	c	0.090	0.005
Effect on mediator	a	0.078	0.0001
Unique mediator effect	b	0.106	0.0005
Indirect effect	ab	0.008 (z = 3.48*)	0.0005
Direct effect	c’	0.080	0.01
Sample size	n = 28 sessions, 21,426 trials		

*Sobel’s test

Supplementary Table 3

Regression coefficients and p values for the mediation analysis testing whether neural slowing mediates the relationship between pupil size and the onset of exploration on the next trial. Related to **Figure 5F**.

		$pupil_{t-1}$ → neural $slowing_{t-1}$ → $explore_t$	
		est. coefficient	p value <
Total effect	c	0.095	0.005
Effect on mediator	a	-0.025	0.0005
Unique mediator effect	b	-0.059	0.06
Indirect effect	ab	0.001 (z = 1.69*)	0.05
Direct effect	c'	0.093	0.005
Sample size	n = 28 sessions, 21,200 trials		

*Sobel's test

Supplementary Figure 1

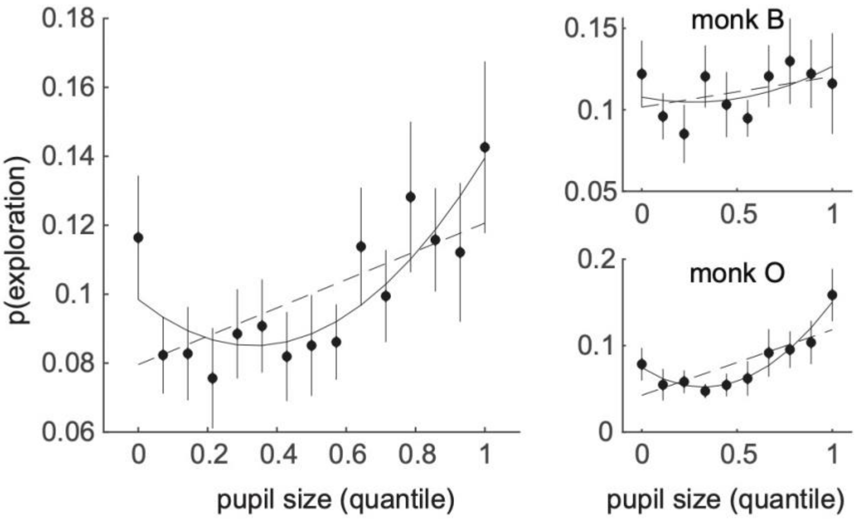

Supplementary Figure 1. Same as **Figure 1E**, but without first explore trials. Error bars depict ± SEM throughout**.**

Supplementary Figure 2

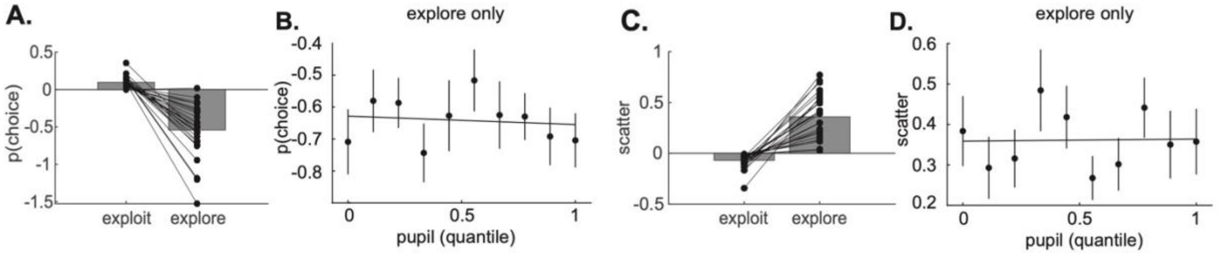

Supplementary Figure 2. Decoded choice probability and scatter index across behavioral states and pupil size. (A) Decoded choice probability (projection onto the correct coding dimension) for exploit and explore states. Dots represent individual sessions, with lines connecting values from the same session across states. (B) Decoded choice probability plotted as a function of pupil size quantile for explore trials alone, related to Figure 4F. (C) Scatter index, a measure of variance in choice-predictive population activity, for exploit and explore states, with lines connecting values from the same session across states. (D) The scatter index plotted as a function of pupil size quantile for explore trials, related to Figure 4G. Error bars depict ± SEM throughout.
